# Neural Network-Based Autonomous Search Model with Undulatory Locomotion Inspired by *Caenorhabditis Elegans*

**DOI:** 10.3390/s22228825

**Published:** 2022-11-15

**Authors:** Mohan Chen, Dazheng Feng, Hongtao Su, Meng Wang, Tingting Su

**Affiliations:** National Laboratory of Radar Signal Processing, Xidian University, Xi’an 710071, China

**Keywords:** bio-inspired model, autonomous search, undulatory locomotion, *Caenorhabditis elegans*, chemotaxis

## Abstract

*Caenorhabditis elegans* (*C. elegans*) exhibits sophisticated chemotaxis behavior with a unique locomotion pattern using a simple nervous system only and is, therefore, well suited to inspire simple, cost-effective robotic navigation schemes. Chemotaxis in *C. elegans* involves two complementary strategies: klinokinesis, which allows reorientation by sharp turns when moving away from targets; and klinotaxis, which gradually adjusts the direction of motion toward the preferred side throughout the movement. In this study, we developed an autonomous search model with undulatory locomotion that combines these two *C. elegans* chemotaxis strategies with its body undulatory locomotion. To search for peaks in environmental variables such as chemical concentrations and radiation in directions close to the steepest gradients, only one sensor is needed. To develop our model, we first evolved a central pattern generator and designed a minimal network unit with proprioceptive feedback to encode and propagate rhythmic signals; hence, we realized realistic undulatory locomotion. We then constructed adaptive sensory neuron models following real electrophysiological characteristics and incorporated a state-dependent gating mechanism, enabling the model to execute the two orientation strategies simultaneously according to information from a single sensor. Simulation results verified the effectiveness, superiority, and realness of the model. Our simply structured model exploits multiple biological mechanisms to search for the shortest-path concentration peak over a wide range of gradients and can serve as a theoretical prototype for worm-like navigation robots.

## 1. Introduction

Biological systems are important inspirational resources for mobile-robot control research. Even the simplest organisms have unique locomotion patterns and remarkable spatial orientation abilities, which depend on their powerful nervous systems. The nematode *Caenorhabditis elegans* (*C. elegans*) has a small, compact anatomy, a fully mapped nervous system comprising only 302 neurons [1,2], and a rich behavioral repertoire; thus, it is an ideal organism for linking neural activity to behavior. ‘*C. elegans* moves in an undulatory fashion by generating sinusoidal dorsoventral bends that propagate from anterior to posterior; the locomotion is involved in most, if not all, of its behavior. Furthermore, *C. elegans* exhibits chemotaxis toward numerous environmental cues, including salt; chemotaxis is the ability to move up (or down) a concentration gradient of a chemical attractant (or repellent). In *C. elegans*, chemotaxis is performed using two parallel strategies [3]: klinokinesis and klinotaxis. Klinokinesis [4] is a biased random walk in which sharp turns occur more frequently in response to a declining (or rising) concentration gradient. The klinotaxis strategy [3] gradually adjusts the movement direction toward the line of steepest ascent (or descent) within the gradient. These behaviors can also be important functions for mobile robots. Chemotaxis-inspired navigation methods can control robots to perform specific tasks, such as chemical leak location [5,6]; radiation measurement [7]; and environment monitoring [8]. Worm-like undulation robots can be deployed in certain special scenarios, such as in pipelines [9] and complex terrain [10,11].

From an engineering perspective, the *C. elegans* chemotaxis behavior is attractive for robotic navigation control. First, this is because the two chemotaxis strategies serve complementary roles to ensure a short search path. Klinokinesis allows the robot to use temporal gradients of environmental variables to quickly correct its direction away from the target, while klinotaxis allows the robot to gradually optimize the path using spatial gradients to align its movement with the steepest gradient direction. Second, *C. elegans* performs chemotaxis only by sensing concentration changes at a single point in its head, suggesting that a robot could mimic the two chemotaxis strategies with a single sensor. This is reasonable, especially for small or resource-constrained mobile robots, as it enables them to make precise steering decisions according to temporal and spatial gradients while carrying a single sensor. Moreover, the clearly delineated nervous system of *C. elegans* provides researchers with the opportunity to study the functional neural circuits [12,13,14] and mechanisms underlying these behaviors [15,16,17,18]. Therefore, these behaviors can be replicated using simple models with efficient biological neural mechanisms, and such models could potentially incorporate these biological methods into robot control applications.

Several studies have explored navigation models inspired by chemotaxis locomotion in *C. elegans*. In early works, researchers [19,20,21] simulated chemotaxis behavior using recurrent networks; hence, they explored computational rules and behavioral strategies for chemotaxis in *C. elegans*. Additionally, Morse et al. [22] designed an autonomous robot to perform chemotaxis-like phototaxis behavior under the control of a simulated neural network. Xu et al. [23] trained dynamic neural networks with single or dual sensory neurons to perform navigation tasks featuring salt attraction and toxin avoidance, mimicking the klinokinesis or klinotaxis strategy; subsequently, those researchers added a speed regulation mechanism to their model [24]. Some studies focused on implementing *C. elegans* chemotaxis-inspired contour tracking by designing spiking neural networks [25,26] and utilizing a neuromorphic processor [27]. However, in the above works, each model was regarded as a point, and the whole-body movement of *C. elegans* was ignored. Deng et al. [28] incorporated the body undulatory locomotion of *C. elegans* into a navigation model emulating chemotaxis for the first time; however, the wave propagation was modeled using an added phase lag term, which was unrealistic, and navigation-induced head deflections could not be propagated. A follow-up study [29] further incorporated the proprioception mechanism [15,30], which is a biological mechanism responsible for the propagation of undulatory waves along the body in *C. elegans*. Demin et al. [31] and Costalago-Meruelo et al. [32] both trained neural circuit models and combined physics engines to simulate chemotaxis and body locomotion in *C. elegans*.

Existing models typically leverage one strategy only. Most (i.e., [19,20,21,22,23,24,25,26,27,28,29,31]) imitate klinokinesis; the model decides to turn or continue straight based on the temporal gradients of the environmental variables only and, therefore, a short search path cannot be guaranteed. Other models (i.e., [23,24,32]) mimic klinotaxis exclusively; they steer in the correct direction depending on the spatial gradient perpendicular to their current path, but usually under the assumption that two sensors are spaced at a certain distance to directly determine the spatial gradient. However, biological studies [14,33] have found that *ASEL* and *ASER* neurons in *C. elegans* are responsible for sensing salt, and function by sensing concentration changes at a single point in the head due to proximity. The state-dependent gating neural mechanism [34,35] indicates that the klinotaxis steering response of *C. elegans* relies on the internal state of the nervous system at the time of the sensory stimulus during undulatory locomotion. Our previous work [17] further identified this mechanism in a neural model based on the *C. elegans* connectome.

Thus, owing to the omission of one chemotaxis strategy, current *C. elegans*-inspired navigation models are often unable to use the temporal and spatial gradient information simultaneously to rapidly search for a gradient source in the environment. In particular, current models typically lack the ability to capture spatial gradient information with a single sensor. To address this problem, we designed a bio-inspired network model that integrates the two strategies (i.e., klinokinesis and klinotaxis) and body undulatory locomotion of *C. elegans* in the context of one sensor. The model exploits the advantages of the complete *C. elegans* chemotaxis behavior, aiming to provide an efficient robotic autonomous search scheme and afford a simple network control prototype for worm-like navigation robots. The proposed model is a multi-joint rigid link system with a network circuit that controls the joint angles. To implement complex behavior in this simple model, we simplified the network circuit as much as possible based on the functional neural circuits of *C. elegans* and incorporated multiple biological mechanisms. The main contributions of this study are as follows.

First, a central pattern generator (CPG) was evolved using a genetic algorithm (GA) to spontaneously generate rhythmic oscillatory signals. Furthermore, a repeating minimal network unit with proprioceptive feedback was designed, which is sufficient to propagate the undulatory wave from anterior to posterior. As such, the model can perform realistic body undulatory locomotion during both forward and navigation-induced steering movements; this behavior was verified through simulation experiments.

Second, dynamic adaptive sensory neuron models were constructed based on the electrophysiological characteristics of the salt sensory neurons in *C. elegans*, which convert information from the sensor into the sensory inputs of the network circuit. Moreover, the state-dependent gating mechanism was incorporated into the model, thereby realizing klinotaxis behavior in the context of a single sensor. Klinokinesis behavior is implemented by fitting a logic function. The model can make steering decisions leveraging the klinotaxis and klinokinesis strategies simultaneously; klinokinesis allows for rapid steering away from the target, while klinotaxis continuously adjusts the movement direction toward the side with the higher concentration. The model was tested in simulations, demonstrating its stable search for environmental variable peaks in directions close to the steepest gradients over a wide range of gradients. The search path was significantly shorter than those of the models employing a single strategy. In addition, the quantitative analysis verified the realness of the two strategies implemented by the proposed model.

The remainder of this paper is organized as follows. Section 2 details the proposed methodology; the overall model architecture and biological basis are first introduced and a detailed description of the sub-network circuits controlling the undulatory locomotion and navigation behavior is then provided. Section 3 presents the experiment results and corresponding analyses, and comparative discussion. Section 4 summarizes the study and suggests future research.

## 2. Methodology

### 2.1. Overall Model Architecture and Biological Basis

**Figure 1:**
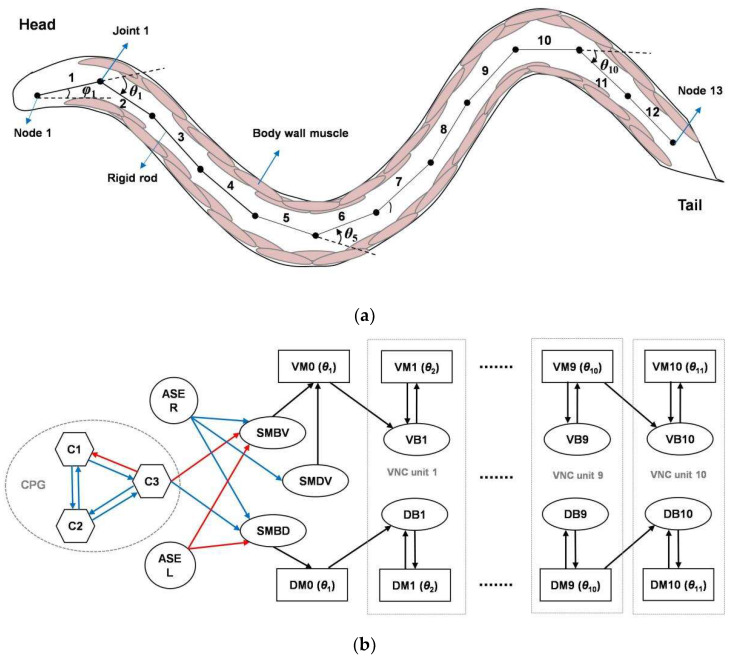
Overall model architecture: (**a**) multi-joint rigid link system abstracted from *Caenorhabditis elegans* (*C. elegans*) structure; and (**b**) network circuit. The circles, hexagons, ovals, and rectangles represent sensory neurons, oscillatory neurons, motoneurons, and muscle cells, respectively. The blue and red lines represent excitatory and inhibitory neural connections, respectively, where the central pattern generator (CPG) connection polarities were determined through evolution and the rest were specifically designed. The black lines represent neuromuscular connections.

The overall model architecture consists of a multi-joint rigid link system and a simple network circuit based on the anatomical structure and functional neural circuits of *C. elegans*, as shown in Figure 1. *C. elegans* is approximately 1 mm long and elliptically cylindrical in shape. It moves on its side on agar and bends in the dorsoventral plane [36], with this movement being mediated by a neuromuscular circuit. Its 95 body wall muscles are arranged along the four body quadrants [37]: the dorsal left, dorsal right, ventral left, and ventral right (DL, DR, VL, and VR, respectively). Each quadrant contains 24 or 23 muscles, which are staggered in pairs. Based on its muscle structure, *C. elegans* was typically modeled with 11 or 12 segments in previous works [28,29]. Similarly, our model contains 12 rigid rods of length *l*, with 11 rotatable joints and 13 nodes, as shown in Figure 1a. Joint 1 controls the head orientation and Node 1 corresponds to the head tip.

The *C. elegans* neural circuits for navigation locomotion are well understood. The motoneurons located in the head and along the ventral neural cord (VNC) drive the dorsoventral muscles to contract and relax rhythmically, generating undulatory locomotion with sinusoidal body waves. Laser ablation studies [13] have shown that, among the five types of VNC motoneurons, the cholinergic B-type excitatory motoneurons are essential for forward locomotion; these include 11 ventral B-type motoneurons (VB neurons) and seven dorsal B-type motoneurons (DB neurons) that form neuromuscular junctions with ventral and dorsal muscle cells, respectively. The four SMB-class and four SMD-class head motoneurons [12] play important roles in regulating the undulatory amplitude and sharp turns, respectively, and innervate the head and neck muscles. In the salt-sensorimotor pathway of *C. elegans*, asymmetric chemosensory neurons (ASEL/R) [14,33] located in the head sense salt stimuli in the environment and communicate with motoneurons through interneurons, inducing head steering. Based on this understanding, we designed a simplified network circuit, the outputs of which control the joint angles of the rigid link system. As shown in Figure 1b, the circuit consists of a head circuit responsible for generating undulations and making navigation decisions, and 10 repeating minimal VNC units responsible for wave propagation. Because this model is two-dimensional, the muscles degenerate to the dorsal and ventral muscles (DMs and VMs, respectively). In addition, the head circuit contains a CPG; however, it is still unclear which neurons belong to the CPG in the head of *C. elegans*.

### 2.2. Definitions

**Figure 2:**
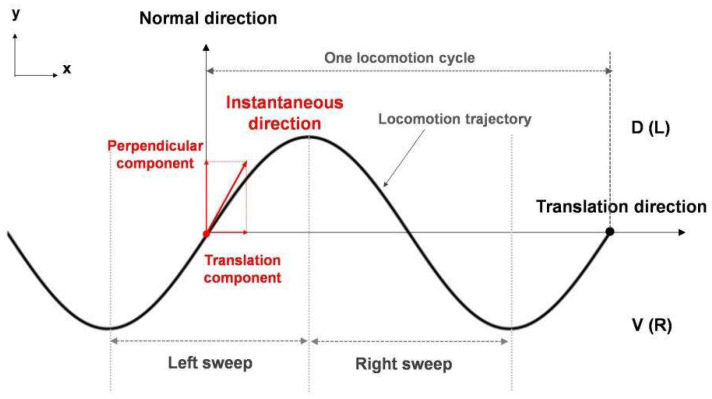
Definitions of kinematics-related terms.

For clarity, some definitions of the kinematics-related terms used in this study are shown in Figure 2. Unless otherwise specified, the position and locomotion trajectory of the model default to those of the head tip (i.e., Node 1 of the rigid link system). Because the model performs undulatory locomotion, the translation direction is the forward direction of the model during movement for one cycle. The normal direction is that 90° counterclockwise to the translation direction. The instantaneous locomotion direction can be decomposed into a translation component and a perpendicular component (the same or opposite to the normal direction). The turning bias is the angle between the current translation direction and the translation direction after one cycle. Sides D and V correspond to the left and right sides, respectively, and a left/right sweep corresponds to half a cycle of movement toward the right/left.

### 2.3. Undulatory Control Circuit

The key to realizing undulatory locomotion is the generation and propagation of an undulatory wave, which requires encoding of rhythmic oscillatory signals in both temporal sequences and spatial patterns. For simplicity, we designed an undulatory control circuit in accordance with the biological view [15,38] that a single CPG produces head-bending waves in *C. elegans*, and that these waves propagate through body from anterior to posterior via proprioceptive feedback. Note, however, that the existence of multiple oscillators in the mid-body VNC motor circuit of *C. elegans* has been suggested [16,39].

#### 2.3.1. CPG

CPGs are neuronal circuits capable of producing rhythmic outputs without rhythmic inputs, typically as a result of reciprocal inhibitory interactions between neurons. Biomimetic CPG controllers are often used in rhythmic motion robots [40,41]. In the proposed model, the CPG consists of three interconnected dynamic neurons (i.e., C1, C2, and C3). In previous models [28,29], a sinusoidal voltage was preassigned to a neuron (i.e., as an oscillator) and the CPG neuron phases were regulated through neuronal interactions. In contrast, we made no explicit *a priori* assumption regarding the way neurons generate oscillations and evolved them to spontaneously generate oscillatory voltages. This mechanism is more in line with biological reality, and from an engineering perspective, the design of a specialized neuron with sinusoidal oscillations is not required.

The neurons in the CPG have first-order nonlinear dynamics, which are expressed by the following first-order ordinary differential equation (ODE):

(1) $$\tau_{i}\cdot \frac{dV_{i}\left(t\right)}{dt}=-\left(V_{i}-E_{i}^{rest}\right)+\sum_{j}w_{i,j}\cdot f\left(V_{j}+b_{j}\right),$$

where $V_{i}\left(t\right)$ denotes the voltage of neuron *i* at time *t*, *τ* is the time constant, and *E^rest^* is the resting potential. The second term on the right is the input current from the other neurons, where $w_{i,j}$ represents the connection weight from neuron *j* to *i*, *b* is the constant bias, and f(⋅) is a sigmoidal function that expresses the nonlinear transmission from the presynaptic neurons to the postsynaptic neurons.

In the proposed model, a simple evolutionary algorithm known as the genetic algorithm (GA) [42] evolves the CPG parameters. There are 15 parameters to be determined: *τ_i_* [0.05, 2]; $w_{i,j}$ [−10, 10]; $E_{i}^{rest}$ [−10, 10]; *b_i_* [−10, 10] (the values in square brackets are the ranges). The parameters are encoded as a real-valued vector with a range of [−1, 1], which corresponds to one individual. The initial population is composed of 500 random individuals and evolves into a new population generation through crossover, mutation, and selection operations. During crossover, two individuals are randomly selected as parents and a new individual, the child, is obtained through two-point recombination. The child is then mutated by adding Gaussian noise with mean zero and a standard deviation (s.d.) of 0.2 to each element of the vector. During selection, the parent with the lower fitness is selected and replaced with the child. If 300 generations are obtained, or the fitness of the best individual exceeds the threshold, the iteration ends.

The goal of this evolution is for the CPG output neuron C3 to generate a sinusoidal oscillatory voltage with a cycle of $T_{osc}=4\;s$ (approximating the *C. elegans* cycle recorded in biological data [35]). Therefore, the model employs the fitness function *F*, which is the product of multiple components: $F=F_{1}\cdot F_{2}\cdot F_{3}$, in terms of $V_{C3}\left(t\right)$, and

(2) $$F_{1}=1-\left|\frac{1}{T}\cdot \int_{0}^{T}V_{C3}\left(t\right)dt\right|,$$

(3) $$F_{2}=f_{N}\left(\max\left(V_{C3}\left(t\right)\right),-\min\left(V_{C3}\left(t\right)\right)\right),$$

(4) $$F_{3}=f_{N}\left(\frac{1}{T}\cdot \int_{0}^{T}\left|\frac{dV_{C3}\left(t\right)}{dt}\right|dt,\;\frac{4\cdot \max\left(V_{C3}\left(t\right)\right)}{T_{osc}}\right),$$

where *T* is the time length and $f_{N}\left(x,x_{0}\right)=\left(x/x_{0}\right)\cdot \exp\left(1-x/x_{0}\right)$ is a normalization function that reaches a maximum of 1 at *x* = *x*_0_. Here, *F*_1_ is combined with *F*_2_ to reward voltage dynamics with zero mean and with the same positive and negative amplitudes. Hence, the same left–right sweep amplitude is ensured for the undulatory locomotion; that is, the translation direction is straight. *F*_3_ is an oscillatory criterion that encourages the voltage change rate to match that of the ideal sinusoidal function, with reference to the criterion used in [43]. The negative values for these functions are set to zero.

The specific amplitude of the voltage is not specified in the evolution, because the output strength can be tuned by connection weights. In addition, some evolved solutions may exhibit damped oscillations that must be re-evaluated over a longer period. Finally, the best individual with stable oscillation is selected from the multiple solutions evolved by the GA to form the CPG.

#### 2.3.2. Undulation Generation and Propagation

As shown in Figure 1b, the *SMBD* and *SMBV* motoneurons receive antiphase oscillatory inputs from neuron C3, with the same connection weight strengths but opposite algebraic signs. For simplicity, *SMBD* and *SMBV* are modeled as neurons with a linear synaptic transfer function, and their voltage dynamics are expressed by the following ODE:

(5) $$\begin{array}{c} \tau_{SMB}\cdot \frac{dV_{SMBD/V}\left(t\right)}{dt}=-\left(V_{SMBD/V}^{}\left(t\right)-E_{SMB}^{rest}\right)+w_{SMBD/V,C3}\cdot V_{C3}\left(t\right)+\\ \;w_{SMB,ASEL}\cdot V_{ASEL}\left(t\right)+w_{SMB,ASER}\cdot V_{ASER}\left(t\right) \end{array}$$

The symbols have the same meanings as in Equation (1), except that the subscripts refer to different neurons; thus, the definitions are not repeated here. $w_{SMBD,C3}=-w_{SMBV,C3}>0$, and the other *SMBD* and *SMBV* parameters are set to the same values. Here, we default to zero sensory input from ASEL/R; this setting is discussed below.

When affected by oscillatory inputs, *SMBD* and *SMBV* generate oscillatory voltages with opposite phases, which are in turn fed to muscles *DM*0 and *VM*0, respectively, via excitatory neuromuscular junctions. The *DM*0 and *VM*0 activation states are expressed as follows:

(6) $$\tau_{A_{0}}\cdot \frac{dA_{DM0}\left(t\right)}{dt}=-A_{DM0}\left(t\right)+w_{M0,SMB}^{m}\cdot V_{SMBD}\left(t\right),$$

(7) $$\tau_{A_{0}}\cdot \frac{dA_{VM0}\left(t\right)}{dt}=-A_{VM0}\left(t\right)+w_{M0,SMB}^{m}\cdot V_{SMBV}\left(t\right)+w_{M0,SMD}^{m}\cdot V_{SMDV}\left(t\right),$$

where $A_{i}\left(t\right)$ denotes the activation state of muscle *i* at time *t* and $w_{M0,SMB}^{m}>0$ is the neuromuscular junction weight from *SMBD/V* to *D/VM*0. In addition, *VM*0 also receives input from *SMDV* with $w_{M0,SMD}^{m}>0$; this mechanism is related to the navigation function, as discussed below. 

The angle of Joint 1 is controlled by the difference between the nonlinear outputs of *DM*0 and *VM*0:

(8) $$O_{D/VM0}\left(t\right)=f\left(A_{D/VM0}\left(t\right)+b_{0}^{m}\right),$$

(9) $$\theta_{1}\left(t\right)=\omega_{0}\cdot \left(O_{DM0}\left(t\right)-O_{VM0}\left(t\right)\right),$$

where $O_{i}\left(t\right)$ and $\theta_{1}\left(t\right)$ denote the output of muscle *i* and the angle of Joint 1 at time *t*, respectively; and $b_{0}^{m}$ and $\omega_{0}$ are the output bias and steering coefficient of *D/VM*0, respectively. In this manner, Joint 1 exhibits rhythmic rotation over time under the control of the network outputs. An increase/decrease in $\theta_{1}\left(t\right)$ means that Rod 1 is turning left/right relative to Rod 2 (i.e., counterclockwise/clockwise).

Biologically, local proprioceptive coupling of adjacent body parts in *C. elegans* converts rhythmic motion near the head into bending waves along the body through a cascade of muscle-to-neuron feedback, with B-type motoneurons transducing the proprioceptive signals [15]. Therefore, in the proposed model, Joints 2–11 are controlled by 10 repeating minimal VNC units, each containing a pair of B-type motoneurons and a pair of muscles, where neurons *DBi* and *VBi* (i=1,2,…,10) receive feedback currents from the muscles of their own units and the anterior adjacent units. The voltage dynamics of DB and VB are as follows:

(10) $$\tau_{B}\cdot \frac{dV_{D/VBi}\left(t\right)}{dt}=-\left(V_{D/VBi}\left(t\right)-E_{B}^{rest}\right)-I_{D/VBi}^{shape}\left(t\right).$$

The parameters of all units are the same. Further, $I_{DBi}^{shape}\left(t\right)$ and $I_{VBi}^{shape}\left(t\right)$ are the proprioceptive feedback currents of *DBi* and *VBi*, respectively, and are expressed as follows:

(11) $$I_{DBi}^{shape}\left(t\right)=\sum_{j=0,1}w_{j}^{shape}\cdot f\left(p_{j}\cdot \theta_{i-j+1}\left(t\right)\right)\cdot \left(V_{DBi}\left(t\right)-E_{j}^{shape}\right),$$

(12) $$I_{VBi}^{shape}\left(t\right)=\sum_{j=0.1}w_{j}^{shape}\cdot f\left(-p_{j}\cdot \theta_{i-j+1}\left(t\right)\right)\cdot \left(V_{VBi}\left(t\right)-E_{j}^{shape}\right),$$

where the terms with j=0,1 represent the feedback currents from the considered unit and the anterior adjacent unit, respectively; $w^{shape}$ is the proprioceptive feedback weight; $E^{shape}$ is the reversal potential of the B-type neurons to the feedback inputs; and *p* is a constant coefficient.

Through substitution of Equation (9), Equations (11) and (12) can be rewritten as

(13) $$I_{DBi}^{shape}\left(t\right)=\sum_{j=0,1}w_{j}^{shape}\cdot f\left(q_{i,j}\cdot \left(O_{DM(i-j)}\left(t\right)-O_{VM(i-j)}\left(t\right)\right)\right)\cdot \left(V_{Di}\left(t\right)-E_{j}^{shape}\right),$$

(14) $$I_{VBi}^{shape}\left(t\right)=\sum_{j=0,1}w_{j}^{shape}\cdot f\left(q_{i,j}\cdot \left(O_{VM(i-j)}\left(t\right)-O_{DM(i-j)}\left(t\right)\right)\right)\cdot \left(V_{Vi}\left(t\right)-E_{j}^{shape}\right),$$

where $q_{i,j}=p_{j}\cdot \omega_{0}$ for *i* = 1 and $q_{i,j}=p_{j}\cdot \omega_{1}$ for *i* > 1. Here, $\omega_{1}$ is the steering coefficient of *DMi* and *VMi* (*i* > 1).

Similar to Equations (6), (8) and (9), the voltages of *DBi* and *VBi* (i=1,2,…,10) affect the activation states of *DMi* and *VMi*, respectively, and their output differences control the angle $\theta_{i+1}\left(t\right)$. Thus,

(15) $$\tau_{Am_{i}}\cdot \frac{dAm_{D/VMi}\left(t\right)}{dt}=-Am_{D/VMi}\left(t\right)+wm_{Am_{i},B}\cdot V_{D/VBi}\left(t\right),$$

(16) $$O_{D/VMi}\left(t\right)=f\left(A_{D/VMi}\left(t\right)+b_{1}^{m}\right),$$

(17) $$\theta_{i+1}\left(t\right)=\omega_{1}\cdot \left(O_{DMi}\left(t\right)-O_{VMi}\left(t\right)\right).$$

The angles of all joints have been determined at this stage. The Joint 1 angle is determined by the CPG rhythm, and the angles of the other joints are determined by the outputs of the corresponding VNC units. Influenced by the feedback current, the joint-angle oscillation controlled by the output of each VNC unit is the same as that of its anterior joint angle, but with a certain phase lag; therefore, soon after the anterior rod turns, the posterior rod is forced to turn in the same direction. In this manner, the rigid link system has a sinusoidal wave shape, and the shape changes with time.

### 2.4. Navigation Control Circuit

The proposed model simulates chemotaxis behavior in *C. elegans*, utilizing parallel strategies (i.e., klinokinesis and klinotaxis) to navigate based on information from a single sensor. To develop our model, we first constructed sensory neuron models to process the sensor concentration information. We then designed the navigation control circuit (i.e., the head circuit) to make steering decisions in accordance with a concentration peak search. In the designed circuit, the sensory neurons communicate directly with SMBD, SMBV, and SMDV, which in turn connect to *DM*0 and *VM*0. Hereafter, the chemical concentration is taken as a representative environmental variable.

#### 2.4.1. Adaptive Sensory Neuron Models

Electrophysiological recordings [33] have shown the following characteristics of two salt sensory neurons in *C. elegans*: (S1) *ASEL* and *ASER* in *C. elegans* act as ON and OFF cells, respectively, in response to increases and decreases in salt concentration, respectively. (S2) The *ASEL/R* voltage response peak increases with an increase of the up/down step amplitude of concentration and tends to saturation. Following this characteristic, we constructed *ASEL* and *ASER* models using a simple conductance-based approach.

The *ASEL/R* dynamics is expressed by the following ODE:

(18) $$\tau_{ASE}\cdot \frac{dV_{ASEL/R}\left(t\right)}{dt}=-\left(V_{ASEL/R}\left(t\right)-E_{ASE}^{rest}\right)-g_{ASEL/R}\left(t\right)\cdot \left(V_{ASEL/R}\left(t\right)-E_{ASE}^{ext}\right),$$

where $E_{ASE}^{ext}$ is the *ASEL/R* reversal potential to the external input. Further, $g_{ASEL/R}\left(t\right)$ denotes the *ASEL/R* conductance at time *t*, which is determined by the concentration information. All constant parameters of the *ASEL* and *ASER* neurons are equal.

For *ASEL*, $g_{ASEL}\left(t\right)$ is derived as follows:

(19) $$\left\{ \begin{array}{ll} g_{ASEL}\left(t\right)=g_{\max}\cdot \tanh\left(\frac{a\cdot \left|\Delta C\left(t\right)\right|}{1+b\cdot C_{N}\left(t\right)}\right),\; & if\;\Delta C\left(t\right)>0\\ \tau_{g}\cdot \frac{dg_{ASEL}\left(t\right)}{dt}=-g_{ASEL}\left(t\right),\; & otherwise \end{array}\right.’$$

where $g_{\max}$ is the maximum conductance; $\tau_{g}$ is the delay time constant of the conductance; a,b>0 are two constant coefficients; and ΔC(t)=C(t)−C(t−Δt) represents the concentration difference between two consecutive samples, where C(t) represents the instantaneous concentration detected at time *t* and Δ*t* represents the sampling interval. The hyperbolic tangent function (tanh(⋅)) ensures conductance saturation.

The sensory neurons of *C. elegans* can change their sensitivities to adapt to an environment in which they have lived for some time [44]; however, this characteristic has not been incorporated into previous navigation models inspired by *C. elegans*. In the present model, $C_{N}\left(t\right)$, a term representing the historical average absolute concentration difference over the past *N* s is introduced, which is expressed as follows:

(20) $$C_{N}\left(t\right)=\frac{1}{N}\cdot \sum_{t^{\prime }=t-N}^{t}\left|\Delta C\left(t^{\prime }\right)\right|.$$

This term allows sensory neurons to have an adaptive characteristic: (S3) sensory neurons can adaptively modulate their response sensitivities to gradients according to the local gradient amplitude detected over a recent period; the sensitivity is high when the amplitudes of the recently detected gradient are small; and decreases when the recent gradient amplitudes are large. Thus, the model can operate effectively over a wide range of gradients.

Consequently, when there is no concentration gradient, the *ASEL* conductance is zero by default. When a positive gradient is detected, the conductance becomes positive, activating ASEL; after the gradient disappears, the conductance value gradually returns to zero and the *ASEL* voltage gradually returns to the resting potential ($E_{ASE}^{ext}=0\;\mathrm{mV}$ here).

As regards *ASER*, the dynamic equations of its conductance $g_{ASER}\left(t\right)$ obey a set of similar equations to Equations (19) and (20), with the replacements L→R and ΔC(t)>0→ΔC(t)<0; hence, *ASER* responds only to concentration decreases.

#### 2.4.2. Klinotaxis Control Circuit

According to the state-dependent gating mechanism [17,34,35], klinotaxis in *C. elegans* may depend on the alternating sensitivities to sensory inputs of neural outputs responsible for controlling dorsal and ventral turns during sinusoidal locomotion. We designed the klinotaxis control circuit based on this neural mechanism, in which *SMBD* and *SMBV* receive sensory inputs and coordinate with *DM*0 and *VM*0 to regulate steering. 

**Figure 3:**
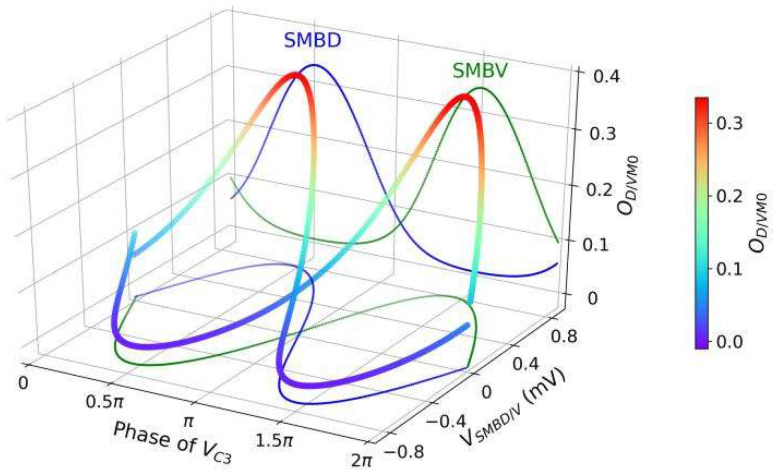
Dynamics of *SMBD/V* motoneuron and *D/VM*0 muscle in the absence of sensory input. The solid-colored curves are the projections of three-dimensional (3D) curves onto each plane.

A negative bias $b_{0}^{m}$ with a large absolute value is set for *DM*0 and *VM*0, and shifts their outputs toward the lower saturation region owing to the nonlinear sigmoidal function (see Equation (8)). Figure 3 shows the dynamics of the klinotaxis control circuit in the absence of a sensory input. The antiphase voltages of *SMBD* and *SMBV* drive the activation states of *DM*0 and *VM*0 to undergo antiphase oscillations, and the outputs of *DM*0 and *VM*0 alternately saturate. During the half period when the SMBD/V voltage is small, the D/*VM*0 output is saturated to zero, where the output is insensitive to the input. As such, *DM*0 and *VM*0 form a state-dependent gating.

In the presence of sensory input and when a concentration increase/decrease is detected during undulatory locomotion, the perpendicular component direction relative to the instantaneous locomotion direction is the side with higher/lower concentration. Therefore, the model should steer in the same/opposite direction to the perpendicular component of the instantaneous direction. This suggests that *ASEL* and *ASER* should have antagonistic effects on the neural output; therefore, we set $w_{SMB,ASEL}<0$ and $w_{SMB,ASER}>0$.

The above two designs ensure two key features of the klinotaxis behavior, respectively: (f1) When the same concentration change is detected during the right and left sweeps, opposite steering is induced (i.e., state dependence), and (f2) when concentration changes with opposite polarity are detected at the same locomotion phase, opposite steering is induced (i.e., sensory dependence).

**Figure 4:**
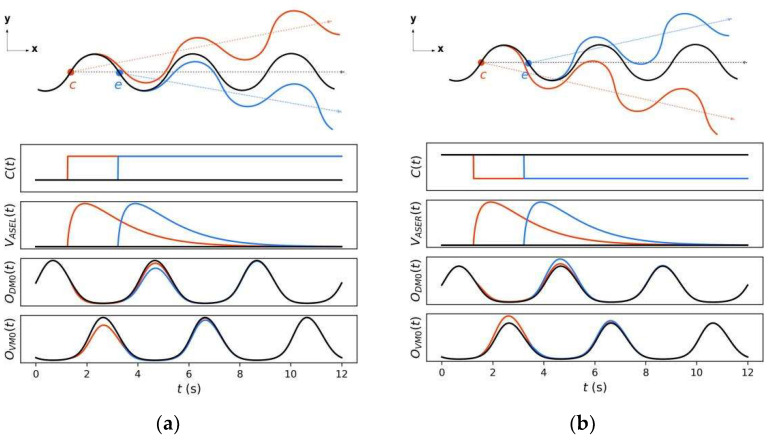
Changes in sensory neuron voltages and muscle outputs (bottom) as well as steering responses (top) caused by application of an up (**a**); or down (**b**) steps at two different locomotion phases. Black, orange, and blue represent the cases where no sensory stimulus is applied and where the sensory stimulus is applied at phase points *c* and *e*, respectively.

**Figure 5:**
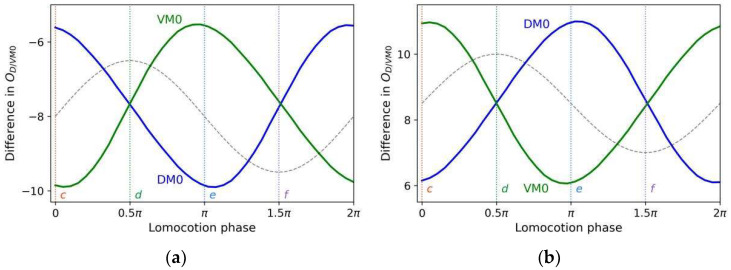
Cumulative differences in *D/VM*0 output induced by application of an up (**a**); or down (**b**) step at each locomotion phase, where phase points *c* and *e*, respectively, correspond to phases *c* and *e* in Figure 4. The black dashed lines represent the shape of the locomotion trajectory.

Figure 4 shows how the control circuit yields a steering decision corresponding to klinotaxis behavior when a concentration increase or decrease (an up or down step, respectively) is detected at two different locomotion phases. The up/down step evokes a continuous voltage response from *ASEL/R* over a certain timescale, which is transmitted by motoneurons; this response yields a subsequent significant decrease/increase in the output of the sensitive muscle and an almost constant output of the saturated muscle. Therefore, the difference between the dorsal and ventral output decreases/increases, causing a decrease/increase in the bending angle of the subsequent undulatory locomotion, which has a duration of approximately half a cycle. As a result, the trajectories are biased toward the same/opposite side of the perpendicular component. Figure 5 shows the cumulative changes in the subsequent outputs of *DM*0 and *VM*0 induced by applying an up or down step at each locomotion phase. When a concentration step is detected at a phase with a larger perpendicular component (such as phases *c* and *e*), the difference between the resulting cumulative differences of the dorsal and ventral muscle outputs is larger, indicating that the steering amplitude is larger. For phases where the perpendicular component is zero (phases *d* and *f*), the difference between the resulting cumulative differences of the dorsal and ventral muscle outputs is approximately zero; therefore, almost no steering is induced. Furthermore, for the right sweep (phases between 0.5π and 1.5π), the change in amplitude of $O_{DM0}$ exceeds that of $O_{VM0}$, and the reverse for the left sweep; that is, the steering is reversed for the right and left sweeps. Consequently, the circuit can correct the model direction according to gradients in the normal direction throughout the locomotion; hence, the model steers to the side with the higher concentration.

#### 2.4.3. Klinokinesis Control Circuit

In the proposed model, steering mimicking klinokinesis behavior is mediated by an *SMDV* motoneuron, the dynamic equation of which is shown below.

(21) $$\tau_{SMDV}\cdot \frac{dV_{SMDV}\left(t\right)}{dt}=-\left(V_{SMDV}\left(t\right)-E_{SMDV}^{rest}\right)-w_{SMDV,ASER}\cdot f\left(V_{ASER}\left(t\right)\right)\cdot \left(V_{SMDV}-E_{SMDV,ASER}\right),$$

where $w_{SMDV,ASER}$ and $E_{SMDV,ASER}$ are the connection weight from *ASER* to *SMDV* and the corresponding reversal potential, respectively.

**Figure 6:**
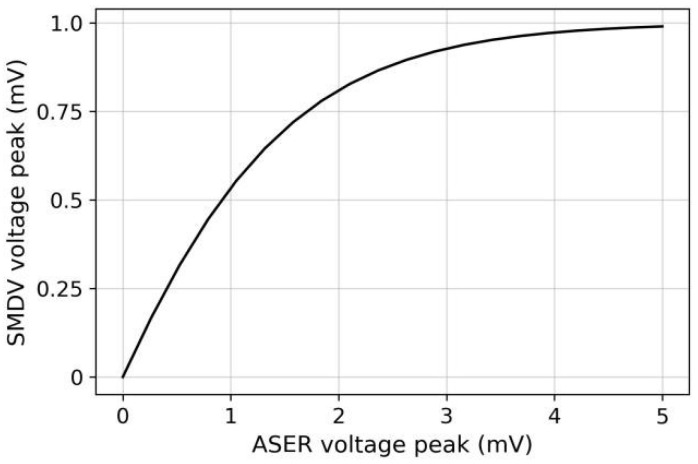
Logic function for mimicking klinokinesis behavior.

In this study, the *SMDV* response voltage was designed to fit the logic function shown in Figure 6 through the parameter settings. This approach is similar to that in the literature [29]. The klinokinesis-related steering depends on the negative temporal gradient; therefore, the *SMDV* response voltage is a function of the *ASER* voltage. When a positive gradient is detected, *ASER* has no sensory response ($V_{ASER}=0\;\mathrm{mV}$). This implies that the model is moving toward the increased concentrations and the model therefore does not need to turn. Therefore, *SMDV* does not generate a voltage response ($V_{SMDV}=0\;\mathrm{mV}$) and does not transmit a turning signal to *VM*0. However, when a negative gradient is detected, the *ASER* response voltage activates *SMDV*, which in turn increases the activation state and output of *VM*0 (see Equation (7)); thus, the model turns right. The greater the deviation between the movement direction and direction of the concentration peak, the larger would be the amplitude of the negative gradient detected and the stronger would be the extent to which the model should correct the direction; accordingly, the *SMDV* voltage response should be large to send a large turning signal. Therefore, the *SMDV* voltage amplitude is proportional to that of the *ASER* voltage and tends to be saturated, enabling the model to correct the direction according to the deviations from the peak direction while preventing oversteering.

## 3. Simulation Results and Discussion

To verify the effectiveness of the proposed model, we tested its undulatory locomotion behavior without sensory input and its autonomous search behavior under simulated scenarios with concentration gradients. Then, we observed the shape of the link system during the steering behavior and quantitatively analyzed the search trajectories. In the simulation experiments, the model was assumed to have l=0.1 mm and a constant-velocity v=0.25 mm/s based on data for real *C. elegans*. Except for the CPG parameters, the parameters in the network circuit were determined by trial and error. All experiments were conducted using Python 3. 8, and the ODEs were solved by Euler integration with a 0.01-s step.

### 3.1. Rhythmic Patterns of CPG and Joint Angles

**Figure 7:**
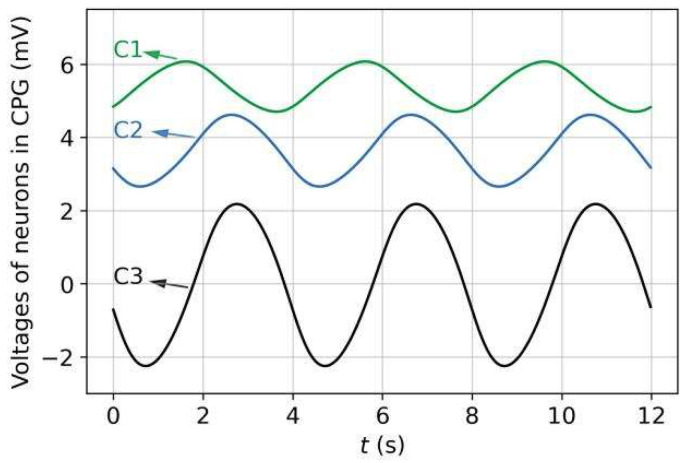
Voltage dynamics of CPG neurons.

**Figure 8:**
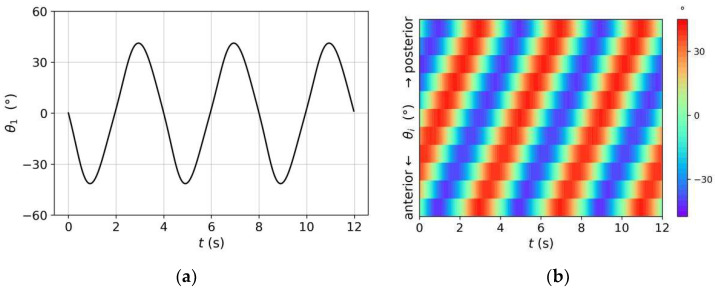
(**a**) periodic changes in Joint-1 angle; and (**b**) kymogram depicting changes in all joint angles over time.

Figure 7 shows the voltage dynamics of the CPG neurons, obtained from our simulations. Through the neuron interactions, the C3 neuron, as the CPG output neuron, generated an approximate sinusoidal oscillatory voltage with a 4-s period and the same positive and negative amplitudes. In the absence of sensory input, the oscillatory voltage of C3 (processed and transmitted by the head network circuit) caused the Joint-1 angle to vary periodically, being centered at zero with the same period, as shown in Figure 8a. Figure 8b shows the changes in all joint angles over time. All joint angles exhibited the same rhythmic oscillation pattern, but the oscillation of each joint (except Joint 1) had a time lag of approximately $T_{osc}/10$ compared with that of its anterior adjacent joint. Through backward propagation, the oscillation of the Joint-11 angle was almost synchronized with that of Joint 1.

### 3.2. Forward Undulatory Locomotion Behavior of Model

**Figure 9:**
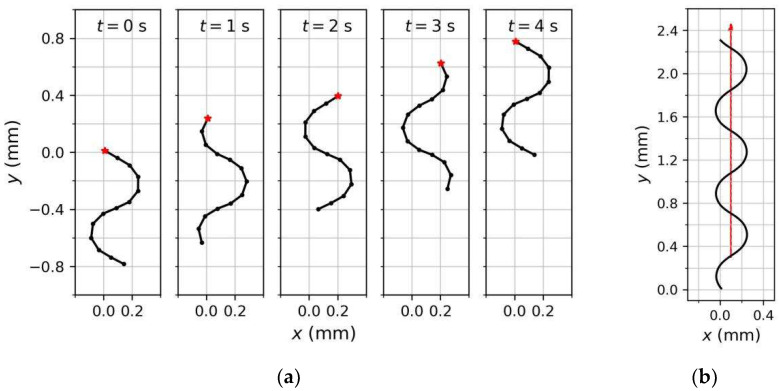
Model shapes in one cycle (**a**); and the forward locomotion trajectory (**b**); under the rhythmic pattern shown in Figure 8. The red line represents the translation direction.

We observed the behavior of the model under the rhythmic pattern shown in Figure 8. Figure 9a shows the positions and shapes of the rigid link system at 1-s intervals during the first period. We initialized the *t* = 0 s position as (0,0) and *φ*_1_(0) = 150°. In terms of spatial mode, *θ*_1_(0) = 0° and the joint angles from front to back increased, decreased, and increased again, thereby shaping the link system as a sinusoidal wave, similar to the real-world behavior of *C. elegans*. As time progressed, Joint 1 rotated periodically and the model moved forward in a fluctuating pattern, forming a sinusoidal trajectory. The posterior joints and, hence, the shape of the link system, varied accordingly. For example, the model was transformed from the left sweep at *t* = 0 s to the right sweep at *t* = 1 s and 2 s. After one period (at *t* = 4 s), the trajectory drew a sine curve with a complete period and the system shape returned to that at *t* = 0 s. The system wavelength during locomotion was approximately 0.83 times the system length (measurements of real *C. elegans* on agar fall in the range of 0.4 to 0.9 [43]). Additionally, from the trajectory in Figure 9b, the model traveled straight with an undulating pattern in the absence of sensory input, because the positive and negative swing amplitudes of *θ*_1_ were consistent (Figure 8a).

### 3.3. Adaptive Responses of Sensory Neuron Models

**Figure 10:**
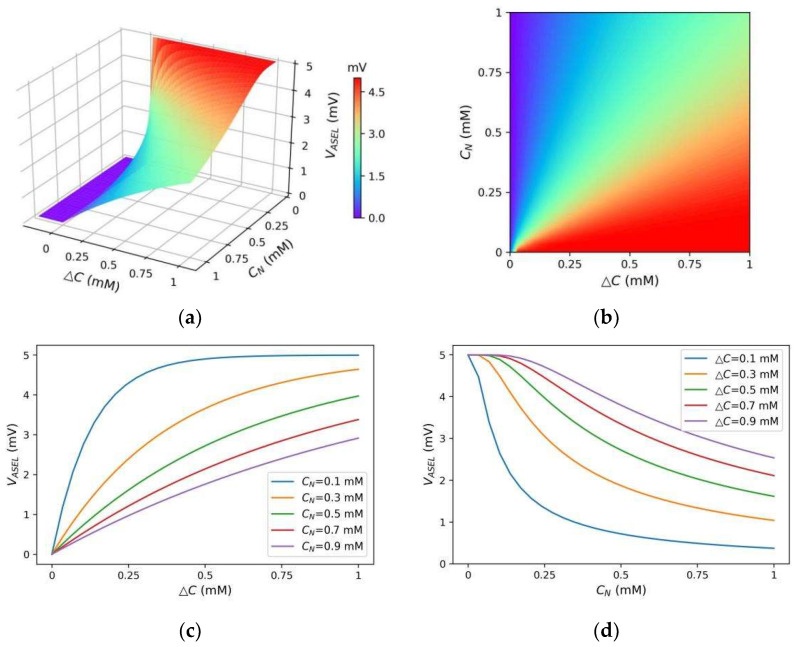
(**a**) *ASEL* voltage peak as function of concentration difference Δ*C*(*t*) and historical average absolute concentration difference *C_N_*(*t*); (**b**) top view of (**a**); (**c**) variation tendency of *ASEL* voltage peak with the change of Δ*C*(*t*); and (**d**) variation tendency of *ASEL* voltage peak with the change of *C_N_*(*t*).

*ASEL* was selected as an example to verify whether the responses of the sensory neuron model met the desired characteristics given in Section 2.4.1. As shown in Figure 4a, a concentration up step evoked *ASEL* depolarization, and its voltage trace exhibited a rapid increase and subsequent slow decay. According to the design of sensory neuron models, the *ASEL* response amplitude is influenced by the concentration difference Δ*C*(*t*) and historical average absolute concentration difference *C_N_*(*t*) (refer to Equations (19) and (20)). Figure 10 shows the *ASEL* response voltage peak as a function of Δ*C*(*t*) and *C_N_*(*t*), from which the following observations can be drawn:

- *ASEL* responded to concentration increases (Δ*C*(*t*) > 0) only, as required in S1;
- When positive Δ*C*(*t*) was small, the *ASEL* response amplitude was small. The *ASEL* voltage peak increased with the increase in Δ*C*(*t*) for any given *C_N_*(*t*) (Figure 10b,c). This indicates that for the local scenario or the scenario with small gradient differences (i.e., the model did not need to re-adapt to the new gradient range), the *ASEL* response amplitude was proportional to the detected temporal gradient, as required in S2. On this basis, the model can control the steering amplitude by comparing magnitudes of the same-polarity gradients;
- When *C_N_*(*t*) was small, the *ASEL* response amplitude was large, implying that *ASEL* was highly sensitive to the gradient. The *ASEL* voltage peak decreased with the increase in *C_N_*(*t*) for any positive Δ*C*(*t*) (Figure 10b,d). This indicates that the *ASEL* response was adaptive to the local environment. When exposed to large concentration gradients for a period, the sensory neurons became less sensitive to the gradients. Thus, the characteristic in S3 was satisfied, allowing the model to operate effectively across a wide range of gradients.

In conclusion, the response dynamics of *ASEL* satisfied the desired characteristics. Similar characteristics were observed for *ASER*, except that the sensory neuron responded to concentration decreases as designed; these results are not reported here.

The responses of sensory neurons reflect only the temporal gradients scaled by *C_N_*(*t*). Klinokinesis-related steering depends solely on the scaled temporal gradient, according to the *ASER* response. Meanwhile, klinotaxis-related steering depends on the normal gradient, i.e., gradients in the normal direction (Figure 2), according to the *ASEL* and *ASER* responses. This is achieved via the state-dependent gating mechanism, that is, the klinotaxis control circuit combines the network internal state with the sensory neuron responses to implicitly extract the normal gradient information corresponding to the current locomotion phase (see Section 2.4.2 for details).

### 3.4. Autonomous Search Behavior of Model

To verify that the proposed model can search for a concentration peak in a direction close to the steepest gradient, we performed simulations with concentrations having Gaussian distributions.

**Figure 11:**
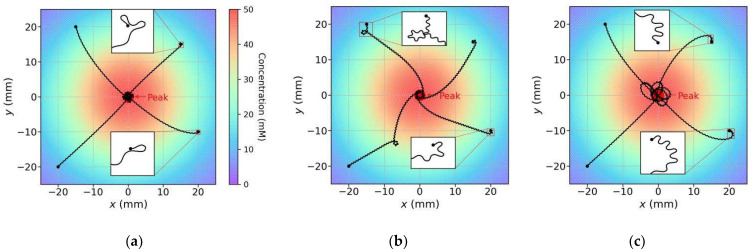
Search trajectories of three models in a given scenario: (**a**) the model exploiting parallel (klinokinesis and klinotaxis) strategies; (**b**) the klinokinesis-only model; and (**c**) the klinotaxis-only model. The four trajectories of each model have different initial positions and orientations, and the initial conditions were the same for all models. The color of each position in the scenario represents the concentration of that position (marked by the color bar in (**a**)).

First, we observed the model trajectories in a scenario where the concentration peak position was (0, 0) and the maximum concentration was 50 mM. The model began moving from different initial positions and with different initial orientations. For comparison, we eliminated one strategy (klinokinesis or klinotaxis) from the proposed model, and the same experiments were also performed for models utilizing a single strategy. The navigation method of the model with klinotaxis eliminated is similar to that of the previous models [29]. Figure 11 shows several trajectories for the three models (two parallel strategies, klinokinesis only, and klinotaxis only) obtained for the same initial conditions. Comparison of these trajectories yielded the following observations:

- For various initial conditions, the three models moved forward in a sinusoidal fashion and successfully reached the concentration peak; however, their search trajectories varied;
- As regards the search trajectories of the klinokinesis-only model (Figure 11b), for the positive gradient direction, the model did not steer, even if there was a deviation from the peak direction. The model corrected the locomotion direction by right turns only when negative gradients were encountered; this behavior is consistent with that of the previous models [29]. In other words, the model could only ensure that it was moving close to the peak, but not that it was using a short search path. In such cases, a large locomotion undulation is advantageous, because a large swing facilitates detection of a negative gradient and adjustment to a more favorable direction. However, a large swing also yields a longer path and may increase the search time;
- As regards the search trajectories of the klinotaxis-only model (Figure 11c), this model continuously and gradually veered toward the side with higher concentration throughout the undulatory locomotion. The orientation adjustment of the model was slightly slower than that of the klinokinesis-only model for negative gradients (compare the beginnings of trajectories whose beginning position are (20, −10) in Figure 11b,c). Because the *ASEL* responses reduced the Joint-1 oscillation amplitude when the model moved toward positive gradients, the model swing amplitude decreased; hence, the search paths were shortened;
- Our model, which integrates both strategies, yielded significantly shortened search paths (Figure 11a). Regardless of the initial position and orientation, the model reached a peak in a direction close to the steepest gradient. If the search began in a direction away from the peak, the klinokinesis strategy allowed the model to make sharp turns to rapidly correct the direction. Meanwhile, the klinotaxis strategy allowed the model to gradually optimize the locomotion direction according to the deviation between the instantaneous and peak directions.

Two metrics were used to measure the model search performance: the *arrival rate* and *SSR*. The *arrival rate* is the ratio of the number of times the model reached the concentration peak to the total number of experiments, where the peak point is defined as a circular area within 1 mm of the maximum concentration point in the simulations. The *SSR* is the ratio of the model search time to reach the concentration peak to that corresponding to the shortest path (i.e., the linear distance from the initial position to the concentration peak position).

**Figure 12:**
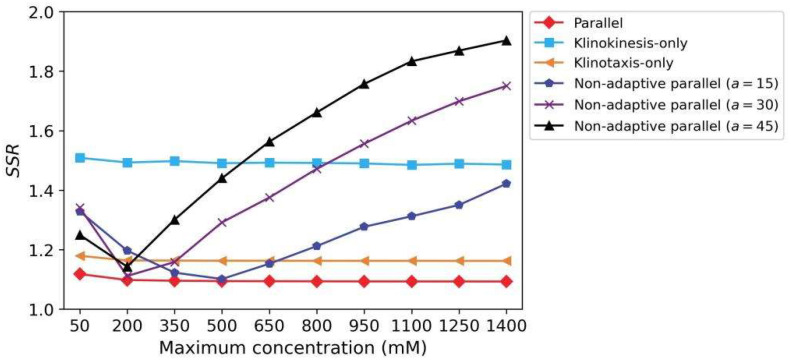
Search performance of all models in scenarios with different concentration gradient ranges. Each point represents the average *SSR* for the model searches, where the model started from different initial positions and orientations.

We then tested the three models in 10 scenarios with different gradient ranges. The maximum concentration for each scenario varied from 50 to 1400 mM. In each scenario, the models began moving from four different initial positions with 10 different initial orientations; that is, each model was run 40 times per scenario for a total of 400 times across all scenarios. Figure 12 shows the average search performance results obtained for the three models in each scenario, and Table 1 lists the average results for the models across all experiments. The following conclusions were drawn:

- The *arrival rates* of all three models were 1, indicating that the models reached the concentration peaks in all experiments regardless of whether they used single or parallel strategies. In addition, the standard deviations of the *SSR*s for all models were very small, indicating that the models had robust search performance for different scenarios and initial conditions;
- Compared with the models using a single strategy, the average *SSR* of the model using parallel strategies was the smallest and close to 1, indicating that the search paths were effectively shortened by simultaneous use of the two complementary strategies, and that the search paths were approximated to the optimal paths although the model moved along a waveform path instead of a straight line;
- The average *SSR* of the klinokinesis-only model was much larger; this was because it did not optimize the path in the direction of the positive gradients and the swing amplitude of the undulatory locomotion was large (see Figure 11b).

Additionally, to verify the role of dynamic adaptation in the sensory neuron models, we conducted the same experiments on a non-adaptive model. In that case, the sensory neuron models did not contain the normalization term with respect to *C_N_*(*t*). That is, the *ASEL* conductance activation term in Equation (19) was modified to $g_{ASEL}\left(t\right)=g_{\max}\cdot \tanh\left(a\cdot \left|\Delta C\left(t\right)\right|\right)\;$, and the corresponding *ASER* term was similarly modified. The coefficient, *a*, took on three different values. Comparison of the results presented in Figure 12 and Table 1 reveals that the non-adaptive models had comparable search performance to that of the adaptive model within a narrow gradient range, and that the search performance deteriorated seriously beyond this range. For small *a* in particular, the response amplitudes of the sensory neurons in the small-gradient scenario were too small, which may have caused a model search failure (i.e., the *arrival rate* was not 1). In contrast, the adaptive sensory neuron models could dynamically adjust their response sensitivities to the gradient according to the gradient amplitude in the preceding time period, so that the model maintained stable search performance in all scenarios.

### 3.5. Analysis of Search Behavior

**Figure 13:**
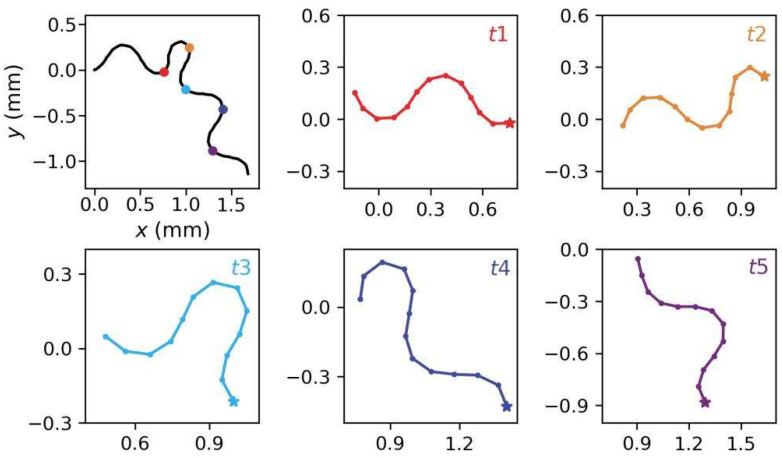
Shape of rigid link system during steering. The upper left diagram shows the model trajectory, and the shapes of the rigid link system at the position indicated by the five colored points are shown in the similarly colored sub-graphs. The pentagram represents the head tip (i.e., Node 1 of the system).

The shape of the rigid link system during the steering induced by the navigation behavior was observed. Figure 13 shows shapes at five different stages as the model underwent an Ω turn (a sharp turn performed by *C. elegans*). When the head turned, the rigid link system bent accordingly (i.e., at times *t*2, *t*3, and *t*4). The link system shape at each stage was close to the corresponding trajectory shape. After the head returned to a normal swing, the entire link system returned to the normal sinusoidal undulation (i.e., at time *t*5). This suggests that the rigid link system could be shaped similarly to the body of a real worm during steering.

**Figure 14:**
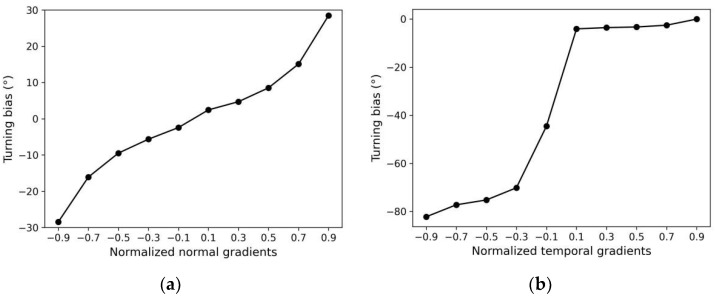
Quantitative analysis of search behavior strategies: (**a**) turning bias vs. Normal concentration gradient of klinotaxis trajectories; and (**b**) turning bias vs. temporal gradient of klinokinesis trajectories. All gradients were linearly normalized to between −1 and 1.

Additionally, to verify that the navigation decisions generated by the model conformed to the two chemotaxis strategies of *C. elegans*, we performed a quantitative analysis of all trajectories of the models using klinokinesis only and klinotaxis only. First, we calculated and statistically analyzed the relationship between the turning biases and normal concentration gradients in the klinotaxis-only trajectories; the result are shown in Figure 14a. The average turning bias was positively correlated with the normal gradient, which is consistent with the statistical results of biological experiments [3]. This suggests that the proposed model mimicked the klinotaxis behavior of real-world *C. elegans*, steering to the side with the higher concentration. We then calculated the relationship between the turning bias and the temporal gradient of the concentration in the klinokinesis-only trajectories, as shown in Figure 14b. For positive gradients, the turning bias was almost zero. For negative gradients, the turning bias was negative (i.e., the model turned right) and the amplitude was proportional to the gradient amplitude. These results have the same trend as biological results [3,4]. In addition, comparing Figure 14a,b, the steering amplitude obtained for the klinokinesis behavior was greater than that for the klinotaxis behavior; this is also consistent with biological findings [3].

### 3.6. Discussion

This section highlights the differences and advantages of our study compared with previous related research. Most existing navigation models inspired by *C. elegans* chemotaxis aim to realize the chemotaxis behavior and, on this premise, explore the underlying mechanisms from the biological perspective; therefore, less attention is paid on the search performance of models from an engineering perspective. In contrast, the purpose of this study is to replicate the complete chemotaxis behavior of *C. elegans*, including parallel chemotaxis strategies and body movement, in the context of one sensor, and to provide an easy-to-implement and good-performance model for worm-like robot navigation control. Table 2 lists the capabilities and properties of related navigation models in the literature and those of the proposed model, which are summarized as follows:

(1) Previous models typically adopted one strategy to perform navigation tasks. In contrast, our model combines two strategies to improve the search performance;
(2) Our model can realize the klinotaxis behavior with a single sensor by incorporating the state-dependent gating mechanism, whereas previous models that mimic klinotaxis typically require two sensors to obtain the required spatial gradient;
(3) Our model can realize body undulatory locomotion during steering by incorporating a proprioceptive mechanism, similar to the model in [29]. However, the structure of our model is simpler;
(4) Our model exhibits adaptive sensitivity to the concentration gradient to cope with scenarios with various gradient ranges, a function which is absent in the previous models.

Additionally, in simulations, the proposed model exhibited realistic undulatory locomotion and a stable search for the shortest-path concentration peak over a wide range of gradients. Moreover, the simulation results have demonstrated that the search performance of our model combining two strategies is significantly better than that of models using a single strategy.

**Figure 15:**
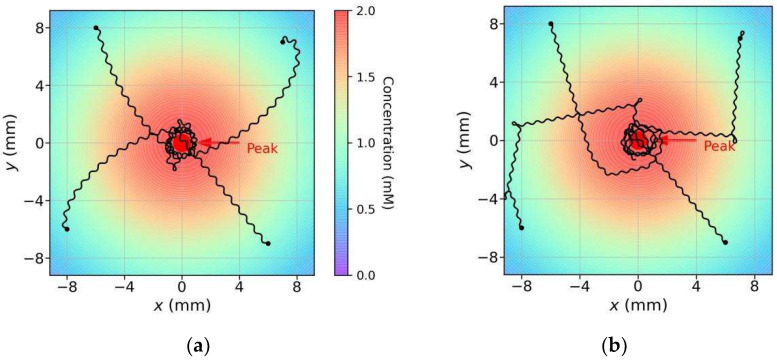
Search trajectories of our model (a); and the reproduced model (b) in the scenario used in [29].

For further comparison, we conducted a comparative experiment between our model and the model in [29]; this model also uses a single sensor and incorporates undulatory locomotion of the body, and the implementation approach of klinokinesis in our model is similar to that of this model. We reproduced the model in [29] using the original logic function and parameters. We tested our model against this model according to the scenario used in [29]; the peak concentration and diffusion range in this scenario are very small. Figure 15a,b show the search trajectories of our model and the model from [29], respectively. The trajectory patterns were consistent with those shown in Figure 11a,b, respectively. Multiple experiments were conducted with four different initial positions and 10 different initial orientations; the average *SSR*s were 1.961 for the reproduced model and 1.486 for our model. The ratio of average *SSR* of the reproduced model to that of our model was 1.32, which was close to the results in Section 3.4; as shown in Table 1, the ratio of average *SSR* of the klinokinesis-only model to that of our model was 1.36. In addition, the reproduced model needed to normalize the concentration gradient in the scenario in advance, while, in practice, it is difficult to obtain *a priori* knowledge of the gradient range of an unknown scenario.

## 4. Conclusions

To incorporate new biological methods into mobile-robot control, a neural network-based autonomous search model with undulatory locomotion inspired by *C. elegans* was proposed in this study. The developed model is the first to simultaneously mimic the body locomotion and the klinokinesis and klinotaxis strategies of *C. elegans* with a single sensor, to search for environmental variable peaks in directions close to the steepest gradients. Multiple biological outcomes are incorporated into the model so that the simple structure is sufficient to achieve complex *C. elegans*-like behavior. Specifically, the CPG and proprioceptive mechanism of *C. elegans* are incorporated in the model to achieve undulatory locomotion, as well as the electrophysiological characteristics of salt-sensory neurons and the state-dependent gating mechanism; the latter is included to achieve klinotaxis behavior in the case of a single sensor. In addition, klinokinesis behavior is realized by fitting a logic function. In this study, the effectiveness and realness of the proposed model were demonstrated through simulation experiments. The model exhibited stable search performance across a wide range of gradients and outperformed models using a single strategy, while exhibiting realistic body undulation.

In summary, the developed model constitutes a simple bio-inspired network control prototype for worm-like navigation robots. In future work, extending the model by including more navigation strategies will contribute to addressing possible problems in complex scenarios, such as lack of gradient or local extremum. Additionally, the developed model will be considered for application in actual robots.

## Figures and Tables

**Table 1:** Search performance results for different models across all experiments (±standard deviation).

Strategies		*Arrival Rate*	Average *SSR*
Klinokinesis only		1.0	1.4922 ± 0.07309
Klinotaxis only		1.0	1.1642 ± 0.09253
Parallel (klinokinesis & klinotaxis)	Adaptive	1.0	1.0964 ± 0.05162
	Non-adaptive (*a* = 15)	0.8	1.2474 ± 0.32701
	Non-adaptive (*a* = 30)	1.0	1.4388 ± 0.36184
	Non-adaptive (*a* = 45)	1.0	1.5720 ± 0.43820

**Table 2:** Comparison of capabilities and properties of models in the literature and in our study (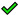 represents the presence of this capability or property in the model).

Articles		Capabilities			Properties	
		Klinokinesis	Klinotaxis	Body Undulatory Locomotion	Single Sensor	Adaptive Sensitivity to Gradients
Xu et al. [23,24]	Model 1	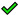			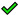	
	Model 2		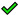			
Santurkar et al. [25]		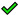			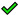	
Shukla et al. [26]		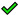			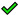	
Kishore et al. [27]		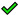			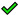	
Costalago-Meruelo et al. [32]			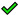	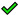		
Deng et al. [28]		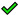		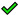	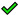	
Deng et al. [29]		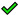		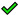	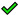	
Our study		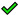	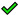	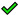	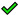	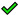

## Data Availability

Not applicable.
